# Mycotoxins evaluation of total mixed ration (TMR) in bovine dairy farms: An update

**DOI:** 10.1016/j.heliyon.2024.e25693

**Published:** 2024-02-06

**Authors:** Daniela Martins, Ana Lemos, João Silva, Marta Rodrigues, João Simões

**Affiliations:** a Department of Veterinary Science, Animal and Veterinary Research Centre (CECAV), Associate Laboratory for Animal and Veterinary Sciences (AL4AnimalS), School of Agricultural and Veterinary Sciences, University of Trás-os-Montes e Alto Douro, 5000-801, Vila Real, Portugal; b Animal Nutrition, DSM-Firmenich, the Netherlands; c CapêloVet, Lda, 4755-252, Barcelos, Portugal; d Vetlima SA, 2050-501, Azambuja, Portugal

**Keywords:** Composite feed, Dairy cows, Emerging mycotoxins, Mycotoxin co-contamination, Silage

## Abstract

The total mixed ration (TMR) is currently a widespread method to feed dairy cows. It is a mixture of raw fodder and concentrate feed that can be contaminated by several mycotoxins. The main aim of this paper was to provide a critical review on TMR mycotoxin occurrence and its usefulness to monitor and control them on-farm. Aflatoxins, zearalenone, deoxynivalenol, T-2 toxin and fumonisins (regulated mycotoxins) are the most prevalent mycotoxins evaluated in TMR. Nonetheless, several emerging mycotoxins represent a health risk at the animal level regarding their prevalence and level in TMR. Even when measured at low levels, the co-occurrence of mycotoxins is frequent and synergistic effects on animal health are still underevaluated. Similar to the animal feed industry, on-farm plans monitoring mycotoxin feed contamination can be developed as a herd health management program. The estimated daily intake of mycotoxins should be implemented, but thresholds for each mycotoxin are not currently defined in dairy farms.

## Highlights

• Evaluation of mycotoxin contamination in total mixed ration (TMR) is a new approach for bovine dairy farms.Double and multiple mycotoxin co-contaminations are highly frequent in TMR contents, even at low levels.
• TMR mycotoxin contamination varies between countries and dairy farms.
• Emergent mycotoxins represent a large component of TMR contamination but are still underestimated in dairy farms.
• The estimated daily intake of mycotoxins (μg/kg body weight/day) is a useful tool to detect animals at risk.
• Double and multiple mycotoxin co-contaminations are highly frequent in TMR contents, even at low levels.

## 1 Introduction

Since the route of mycotoxin intoxication is the consumption of feed, it is important in each animal production system to know the origin of the raw materials and their processing and storage conditions. The total mixed ration (TMR) is the predominant method of feeding high-producing dairy cattle, in which there is the use of different proportions of silage (the main food), raw materials, byproducts, e.g., cottonseed (*Gossypium hirsutum* L.), grains, protein supplements, vitamins and minerals.

Usually, a formula with a great heterogeneity of ingredients is defined, with fodder and feed concentrate being the main components of the diets of these animals [1]. These materials may be potential sources of mycotoxins; therefore, it is extremely important to identify which ingredients are used in the formulation of TMR samples and which contribute to the total load of mycotoxins [2]. Thus, the total dietary exposure of animals to mycotoxins will depend on the composition of the diet and the daily intake of dry matter [3].

Normally, the animal feed industry adopts internal plans for, at least, regulated (legislated) mycotoxins, trying to ensure the safety of their products [4]. Aflatoxins (AF), found in cereal grains, maize gluten, soybean products, sunflower seeds, and cotton seeds; zearalenone (ZEA) and fumonisins (FB), found in maize and maize-derived products; and, deoxynivalenol (DON), ochratoxins (OT) and ergot alkaloids, found in cereal grains, are the main contaminants [5]. Moreover, co-occurrences of mycotoxins are frequent in cereal grains [6]. Awapak et al. [7] reported that all feed concentrates samples (n = 33) collected from Thai dairy cow farms were contaminated by ZEA (average levels = 24.3 μg/kg and maximum levels = 55.6 μg/kg) and flavoglaucin (average levels = 127.8 μg/kg and maximum levels = 859.2 μg/kg). Beauvericin (BEA), FB1, FB2, enniatins (ENN) B and B1, averufin, 3-nitropropionic acid, methylsulochrin, nigragillin, phenopyrrozine, sterigmatocystin, equisetin, siccanol, and several *Alternaria* and *Penicillium* metabolites were also detected in at least 50% of the samples. These findings highlight the frequency of mycotoxin co-occurrences as well as the importance of establishing feasible internal plans to manage mycotoxin hazards in feed concentrate.

Similar internal plans to control mycotoxin occurrence are not consistently provided for fodder (e.g., silages, hay), which is mainly produced by farmers and can represent a relevant source of contamination [8]. The presence of fungi in silage and other fodder (e.g., hay) is related to nutrient and dry matter losses, mycotoxin production, reduced palatability and food intake [9], compromising milk production, animal health and welfare in dairy farms.

A great deal of scientific literature related to mycotoxins in feed regarding cattle has been published, as can be found on the Web of Science. Nonetheless, only a small percentage of recent studies evaluated mycotoxins in TMR feeding systems. This review mainly aimed to provide an overview of mycotoxin occurrence in TMR feeding systems and related factors that influence their prevalence and level, as well as those provided by silage, the main TMR ingredient. On-farm monitoring procedures for mycotoxin control were also addressed.

## 2 Concepts of mycotoxins and current context in dairy cattle

Mycotoxins comprise a large variety of low molecular weight compounds, most of which are >1000 Da. They are produced mainly by the secondary metabolism of certain filamentous fungi. The most common toxicogenic species belong to the genera *Fusarium*, *Aspergillus, Penicillium* and *Alternaria* [10]. More than 300 to 400 mycotoxins have been identified and reported in several studies, but only a few have been considered of economic concern and health hazards. The most relevant of those mycotoxins are AF, FB, OTA, trichothecenes (TRC) and ZEA [11]. Another significant issue to be taken into consideration is the possible presence of modified mycotoxins that are derived from regulated mycotoxins, whose structure has changed by natural metabolization processes and because of the processing of food and feed [12]. These mycotoxins are found in plants, resulting from plant defense reactions to fungal infection, or produced by the fungus itself. They are usually formed by conjugating the original mycotoxin, the predominant pathway being glycosylation, sometimes followed by subsequent metabolic steps to facilitate its biotransformation [13]. Some examples of modified mycotoxins are deoxynivalenol-3-glucoside and zearalenone-14-glucoside [14,15]. These modified mycotoxins are often not detected when analyzing the mycotoxins of the plants from which they originate due to changes in the physicochemical properties of their molecules [16]. They are commonly called “masked” mycotoxins, and it is essential to develop analytical methods that allow their identification and quantification [15]. The occurrence of masked toxins will increase the overall burden and toxicity of mycotoxins [17].

However, the presence of fungi in the diet of dairy cattle does not necessarily mean that there is contamination by mycotoxins and vice versa [18]. The level of mycotoxins can occur independent of the growth of fungi (primary metabolism) [19,20]. Normally, mycotoxins are produced at the end of the fungal growth phase [21].

These occurrences depend on silages being produced under suitable conditions; when this does not happen, more diverse fungal contamination can occur. The growth of these fungi is influenced not only by temperature and humidity but also by mechanical damage to plants (e.g., insects, birds) or storage conditions. In the case of silages, it is influenced by the quality of the raw material, additives, silo filling rate, and storage watertightness to reduce oxygen [19].

Mycotoxins are a threat to human and animal health [22]. Animal consumption of mycotoxin-contaminated crops may cause adverse health effects. Different types of toxic effects can be observed depending on the individual mycotoxins and their dose intake over time, i.e., (sub)acute versus chronic mycotoxicosis. Reduced feed intake and milk yield are common signs observed in the presence of several mycotoxins (e.g., AF, ZEA, DON, OTA, T-2 toxin, FB). Impaired immunity, reproductive alterations, hepatotoxicity, gastroenteritis, nephrotoxicity, chronic to acute disease, and even death are other prominent adverse effects (see the review by Kemboi et al. [23]).

AF can negatively impact the production, immune health, and reproduction of ruminant animals [24]. FB are mycotoxins that are cytotoxic, hepatotoxic and nephrotoxic, and can be absorbed in the gut [25]. The mycotoxin DON can cause numerous problems in animals, including gastrointestinal disorders, soft stools, immunodepression, and a general decrease in performance due to feed refusal [26]. ZEA is a mycotoxin that has an estrogenic effect that leads to disturbance of the estrous cycle, pathological changes in the reproductive tract of males and females, decreased fertility, decreased neonatal survival in females and offspring, and impaired spermatogenesis [27].

The economic impact of mycotoxins in dairy farms is primarily related to income loss due to a decrease in milk production and poses threats to public health due to milk contamination (e.g., AFM1), which causes its rejection in the milk trade [23,28]. Nonetheless, costs associated with increased reproductive alterations, incidence of diseases, morbidity and mortality of cows, veterinary care, and elimination of contaminated feed can also be determinants [29,30]. According to EU law [31], only some mycotoxins are regulated in some feed materials, e.g., AFB1 (threshold of 5 μg/kg dry matter), FBB1 + B2 (20,000 μg/kg), DON (2000 μg/kg), ZEA (500 μg/kg), OTA A (250 μg/kg) [32], and T-2 toxin + HT-2 toxin (250 μg/kg) [33]. Nonetheless, the co-occurrence of mycotoxins in TMR below the guidance values can cause adverse defects in animal performance and health [34].

Currently, there is also growing concern about emerging mycotoxins, of which there is not enough toxicological data for regulation and maximum acceptable levels that protect humans and animals; however, there has been a rapid increase in their incidence [17,35]. The name “emerging mycotoxins” refers to a group of chemically modified mycotoxins that have no (EU) legislation thus far [36]. The most common emerging mycotoxins of *Fusarium* are ENN, BEA, moniliformin (MON), fusaproliferin (FP), fusidic acid (AF), culmorin (CUL), and butenolide (BUT) [4,17]. A great number of emerging mycotoxins and their co-occurrence assume high relevance in the feeding systems of dairy cattle. Penagos-Tabares et al. [37] detected 20 different emergent mycotoxins in Austrian dairy farms, mainly derived from the genera *Fusarium* (n = 15) but also from A*lternaria* (n = 3), *Aspergillus* (n = 1), and *Penicillium* (n = 1). High co-occurrences (>70%) between them, regulated mycotoxins, and other fungal derivate metabolites were observed.

Several methodologies to identify and quantify mycotoxins in feed samples from dairy farms have been developed over the past decades (see review by Janik et al. [38]). They include chromatographic and immunochemical-based methods. We highlight the development of liquid chromatography with tandem mass spectrometry (LC-MS/MS) [39,40], which is able to quantify hundreds of mycotoxins, including emerging and masked mycotoxins, as well as other fungal metabolites, from different feed matrices in a short period of time. The current expansion of this multi-mycotoxin methodology, at reasonable costs, can provide a useful and accurate tool to detect and quantify the co-occurrence of mycotoxins such as TMR and raw materials such as silages. This aspect is relevant for providing a multi-mycotoxin risk assessment in dairy farms, other than the regulated mycotoxins.

### 2.1 Silages

In many cases, maize silages (the most commonly used silage in dairy) are stored for 14 months or even longer, and over this storage period, there are significant variations in their microbiota [41]. According to Storm et al. [42], the risk of fungal contamination, as well as the growth of filamentous fungi, is higher in the first 5–7 months and lower after 11 months of silo opening (aerobic exposure). Additionally, the amount of yeast and lactic acid bacteria decrease over storage time. Nonetheless, several fungal communities (and their toxins) can persist under adverse environments (low pH, high Co_2_, and low O_2_) [43]. Moreover, positive correlations (r = 0.70–0.99) between silage temperature above ambient temperature and fungal communities have been observed, resulting in aerobic deterioration after silo opening [44]. Usually, the silage temperature follows the trend of air temperature; however, during winter, the silage temperature may be higher than that of air due to the microbial activity present in silage [42,[45], [46], [47]].

**Fig. 1:**
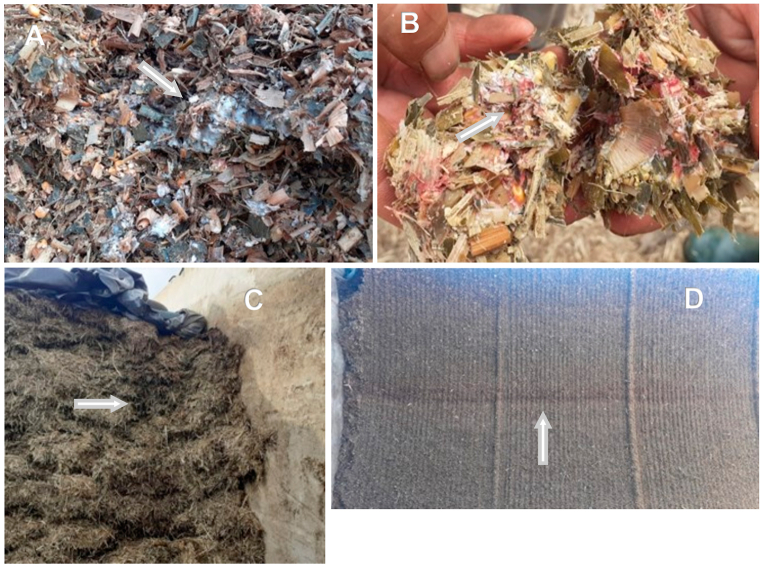
Fungal contamination of maize silages. Legend: White molds (A and B); vertical (C) and horizontal (D) brownish-red stripes into two horizontal trench silos. Beauvericin, enniatin, fumonisin B1 and B2, zearalenone, and deoxynivalenol were detected in these four dairy farms.

Fungal growth is often visible when opening the silo (Fig. 1A and B), being represented by layers approximately 20–80 cm below the surface (blue, orange, green, white, or red color), agglomerates, the size of handles, and/or discolorations of the surface (Fig. 1C and D). When moldy silage is visible, it should be removed, as well as part of the enveloping non-moldy silage, before being supplied to the animals. When the rate of use of silages is too slow, the growth of fungi can progress from the cutting face to the rest of the silage because the silage is exposed to air up to 1–2 m from the cutting face. To avoid the harmful effects of air, the design of the silos and the rate of use should allow a rapid advance in the cost of the silo before there is fungal growth [48].

During the silage process, there is supposed to be a minimization of fungal contamination, as oxygen is consumed toward anaerobiosis and there is a reduction in pH to an acidic condition unfavorable to contamination and the development of fungus [49]. However, if this process is not well carried out and there are bad practices and storage conditions, or even after cutting the silo for feeding the animals, there may be contamination [50]. The moisture content of maize silage also affects the growth of fungi, namely, *Aspergillus* and *Penicillium* (storage fungi) [36,51].

According to Keller et al. [50], there may be variations in the population of fungi and mycotoxins present along the silo, as well as in the different layers (upper, lower, lateral, and central) (see Fig. 1A and C). In addition, much of the maize silage is obtained through annual crops, with a variation in the levels of mycotoxins from year to year. This variation can be caused by temperature and precipitation during the most sensitive periods of grain development or by harvest conditions [35,42,50]. Keller et al. [50] also observed that there was an increase in the total fungal count during the fermentation process of maize silage, as this count was higher in the samples taken after fermentation than in the pre-fermentative step. Contamination by mycotoxins of unfermented feeds, such as cereals, hay, and oilseeds, depends essentially on the pre-harvest conditions and good storage conditions of these foods, while the mycotoxicological quality of fermented feed depends on the technological processing conditions in the change in feed production [[52], [53], [54]].

Maize silage is the main component of milk cow feed in many regions of the world [55]. Maize silage is a significant source of contamination, possibly because maize cobs and leaves are rich in protein and polysaccharides, which can help fungi and other pathogens grow and proliferate [54]. Maize silage is the main source of AFB1, DON, and ZEA [[55], [56], [57], [58]], being DON and ZEA the most prevalent mycotoxins in both maize grain and whole maize [55]. Corn grains, for human and animal feed, have the highest values of FB and, in some regions of the world, of AF [55].

The occurrence of mycotoxins in maize silage samples is higher than that in herb samples [9,36]. Apparently, grass and wheat silages are not a relevant source of ZEA and DON when compared with maize silages [3]. In Austria, Penagos-Tabares et al. [37] observed a higher percentage of maize than grass silages contaminated with ZEA (61% vs. 21%, respectively) and DON (79% vs. 16%, respectively). Moreover, it is possible that the fungal species that contaminate the herb have the ability to produce ZEA but not DON or that the conditions of fungi-contaminated herbs do not favor the formation of DON [3]. In some Finnish regions, BEA (70%) and roquefortine C (49%) were the most prevalent mycotoxins detected, while the prevalence of ZEA was only 8% of a total of 37 grass silage samples [59]. Nevertheless, in Uruguay, Del Palacio et al. [51] detected DON (3000–12,400 μg/kg) in all 220 samples of wheat silo bags. Also, Shimshoni et al. [60] observed ZEA contamination in more than 80% of 30 maize and wheat silage samples. This suggests that regular screening of both ZEA and DON should be performed in wheat silage. In clover grass and lucerne silages, contamination was essentially caused by *Alternaria* mycotoxins and EEN, while complex compound foods had higher contamination of DON, T-2 and HT-2 toxins, ZEA and their metabolites, EEN, FB, *Alternaria* mycotoxins, BEA, roquefortine C, and mycophenolic acid toxins [54].

In temperate regions, high contamination of ZEA and DON can occur in maize silage, which may be due to weather conditions but also to the geographical area and variety of crops and practices used globally [61]. The wetter and warmer Mediterranean climate creates extremely favorable conditions for the growth of molds, which may produce mycotoxins. For this reason, regional differences in mycotoxin development can be observed in the European continent [62].

Since fungal development is very dependent on humidity and temperature conditions [63], current climate changes have a significant impact on the development of toxigenic fungi [64]. Climate change may alter the interactions between the host, its resistance, and the pathogen, thus influencing the production conditions of mycotoxins [65,66]. According to Moretti et al. [62], it is highly likely that there will be an increase in the risk of AF contamination in maize from central and southern Europe in the next 30 years due to the growth-friendly climatic conditions of *Aspergillus flavus*. Moreover, in northern, central, and southern Europe, the profile of mycotoxic species of *Fusarium* in wheat is constantly changing, and the increasing contamination by *F. graminearum* in Central and Northern Europe reinforces this dynamic pattern [62,67].

### 2.2 Total mixed ration

TMR is a mixture of different forages and cereals, whereas silage (maize, grass, and others) and energy-rich concentrate are the main ingredients. They are consumed by dairy cows up to a few hours after processing by an unifeed system. It is expected that the level of mycotoxins in TMR is the sum of their levels in ingredients according to their relative proportion in the composite feed.

Correlations of mycotoxin levels between feed ingredients and composite feed have been reported in poultry (FUM: r^2^ = 0.41, p = 0.03; [68]) and more recently in dairy cows. Moderate Spearman correlation coefficients were observed between concentrate feed and TMR for Ergot alkaloids (ρ = 0.63; p < 0.05), maize stover and TMR for *Fusarium* spp. mycotoxins (ρ = 0.54; p < 0.05), sorghum silage and TMR for FB (ρ = 0.50; p < 0.05), and OAT hay and TMR for ENN (ρ = 0.45; p < 0.05) [51]. These correlations allow us to establish scientific evidence for the degree of contaminated raw feed contribution to mixed rations.

Different feed ingredients of TMR have different potential for mycotoxin contamination. In a recent study performed in northern Italy (2013–2021), Ferrari et al. [69] observed that 5.7% of 10,280 feed samples (cotton, wheat, flour and grain maize, silage maize, high-moisture maize, alfalfa hay, soy, and TMR) exceeded the threshold limit of 20 μg/kg regulated by the EU for AFB1. Nonetheless, the matrices with a higher percentage of AFB1 contamination and presenting the highest contamination values were flour and grain maize (10.1% at total and 1074 μg/kg, respectively) and cotton (5.4% and 728.7 μg/kg, respectively). The percentage of AFB1 contamination (>20 μg/kg) of the remaining feed matrices varied between 0 and 1.6%, despite differences between years. In this study, 2.4% (7/293) of TMR samples were positive for AFB1, but only 0.3% (n = 1) exceeded the limit of 20 μg/kg. Even TMR can present lower levels of mycotoxin contamination than some of the feed ingredients [69], the co-occurrence of mycotoxins from different ingredients [7], and their cumulative toxicity in animals [70].

The same research group [71] reported a parallel pattern of AFB1 contamination between some feeding stuff (>20 μg/kg), namely maize and cotton, and its metabolite AFM1 in raw milk (>50 ng/L) from dairy farms. These raw feed ingredients, which usually present the highest prevalence and values of AFB1, are candidates to serve as markers of contamination for this mycotoxin. Other candidates are high-moisture maize and silage maize. More studies are needed to confirm this hypothesis for AFB1 as well as for other relevant mycotoxins.

The findings of both studies [69,71] emphasized the importance of regular surveillance plants considering different percentages and levels of mycotoxin contamination of raw feed as well as TMR in dairy farms. This realist approach can contribute to ensuring feed and food safety by monitoring both purchased and farm-produced feed. The mixture of feed ingredients allows an increase in double or multiple co-occurrences of mycotoxins in composite feed or TMR [11,72]. Combinations of DON + ZEA (100%), DON + T-2 + HT-2 toxins (97.2%), and ZEA + T-2 + HT-2 toxins (97.2%) have been reported by Twarużek et al. [73]. Moreover, these researchers observed a strong positive correlation between DON and ZEA contents (r = 0.55; P < 0.05; n = 180) and a moderate correlation between DON and HT2 contents (r = 0.41; P < 0.05; n = 180) or even a weak correlation between T2/HT-2 toxins (r = 0.29; P < 0.05; n = 180) [73]. These correlations within mycotoxin contents pose new threats due to their additive, synergic, or combined effects [7,72], even though their levels are lower than the limits established by the EU and national official regulators such as the FDA (US).

Only a few studies (e.g., Refs. [2,9,58,[64], [65], [66], [67], [68], [69], [70], [71], [72], [73], [74], [75], [76], [77]]) have approached the identification and quantification of regulated and emerging mycotoxins in TMR. The majority of them (see Table 1) have been published in the last three years to mitigate this gap and explore the problem of TMR mycotoxin contamination regarding animal health and public health. According to Table 1, the prevalence of different mycotoxins on TMR varies according to the world regions. Most of the levels of regulated mycotoxins are under the limit (threshold) imposed by official regulation for the different feed ingredients. More attention is currently being paid to TMR as the final diet to feed dairy cows, ensuring the control of mycotoxin ingestion throughout the production life of cows. Moreover, the adverse effects on production and animal health imply relevant losses for farmers, especially in countries without specific legislation to control mycotoxins. Mycotoxin co-occurrence, which is more likely expected in TMR than in each feed ingredient, can also contribute to disseminating this new methodology.

**Table 1:** Prevalence and mean level of mycotoxins in total mixed rations to feed dairy cows.

Mycotoxin^c^	Positive Samples	Range (μg/kg)	Mean (μg/kg)	Country; Reference
AF	65.7% (411/626)	2.4–4.9^a^	3.9 (median)	Brazil b; [77]
	8.1% (3/37)	3–5	4.1 ± 1.0 (±SD)	Jordan; [75]
AFB_1_	60.8% (31/51)	1–5	2.4 ± 1.1 (±SEM)	Lithuania; [2]
	36.8% (14/38)	–	0.4 ± 0.8 (±SD)	Spain; [80]
	58.1% (43–74)	≤0.04 ppm	–	Turkey; [74]
ZEA	49.0 (25)	70–700	377.6 ± 249.0	[2]
	24.3% (9)	13–241	71.1 ± 12.5	[75]
	16.0% (31/193)	3–430	90	Spain; [81]
	77.5% (490/632)	37–86^a^	55.2 (median)	b; [77]
	100% (19/19)	4.6–246	38.7 ± 57.2 (±SD)	Mexico; [58]
	50% (236)	–	329 ± 78.6 (±SEM; ppb)	USA; [76]
	21.6% (16)	≤0.031 ppm		[74]
DON	18.9% (7)	0.1–13	2.9 ± 1.8	[75]
	54.9% (28)	50–500	283.9 ± 185.1	[2]
	16.6% (32)	40–790	260.0	[81]
	4.1% (3)	≤0.045 ppm		[74]
	70.3% (422/600)	290–1086^a^	430 (median)	b; [77]
15-ADON	9.3% (18)	9–50	28	[81]
DON-3-Glc	1.6% (3)	10–72	46	[81]
T-2 toxin	29.4% (15)	16–247	106.1 ± 76.0	[2]
	56.8% (21)	9–1626	260.9 ± 78.0	[68]
	31.3 (169/540)	20–54	23.4 (median)	b; [77]
FB	73.0% (27)	333–8623	4396.8 ± 230	[68]
	89%	78.0–5510	1940 ± 1760	[58]
	39.3% (249/633)	370–1190^a^	613.8 (median)	b; [77]
	23%	–	707 ± 279.8	[66]
FB1 + FB2	34.2% (66)	≤1.208 ppm		[81]
	93.2% (69)			[74]
OTA	48.4% (260–537)	4–16^a^	8.3 (median)	b; [77]
	31.1% (23)	≤0.344 ppm	–	[74]
ENN	89%	1.85–37	11.2 ± 9.8	[58]
Type B trichothecenes	89%	78.0–5510	1940 ± 1760	[58]
	98%	–	2604 ± 152.9	[76]

AF–Aflatoxins; ZEA–Zearalenone; DON–Deoxynivalenol; FB–Fumonisins; OTA–ochratoxin A; ENN–Enniatins; ppm–parts per million; ppb–parts per billion.

a 1st quartile- 3rd quartile.

b Dairy and beef cows.

c Positive samples (>20%) of total mixed ration contaminated by metabolites of ergot alkaloids (dihydroergosine), *Alternaria* spp. metabolites (altenuisol, alternariolmethylether, altersetin, tentoxin, tenuazonic acid), *Aspergillus* spp. (averufin, flavoglaucin, phenopyrrozin, seco-sterigmatocystin), *Fusarium* spp. (fusaproliferin, fusaric acid, moniliformin, nivalenol, siccanol, W493), *Penicillium* spp. (7-hydroxypestalotin, bilaid A, citreoviridin, mycophenolic acid, pestalotin, questiomycin and derivates, quinolactacin A) from 19 Mexican dairy farms were reported by Penagos-Tabares et al. [58], and were not reported in the table.

### 2.3 Management of feed mycotoxin contamination

As previously reported, energy-rich concentrates, cereals, or other raw feed provided to farms by feed millers are subjected to internal mycotoxin monitoring plans in most countries. Nevertheless, fodder produced at farms or imported is not routinely analyzed for mycotoxin contamination. For example, it was observed that the increase in straw inclusion increases the levels of FB1, FB2, and *Fusarium*-derived metabolites in mixed rations [33]. Additionally, a positive correlation (ρ = 0.50; p < 0.05) between maize stover and TMR for total mycotoxins was observed [58]. The removal of fodder presenting molds or other abnormal gross appearances is the usual practice in farms to mitigate mycotoxin ingestion. Nonetheless, this procedure is not enough to prevent TMR contamination. In larger farms, it is also common that farmers buy their own raw feed (e.g., brewer’s grains, soya meal, pulp molasses, gluten feed) and do not perform a mycotoxin analysis. For example, Twarużek et al. [73] analyzed 179 suspected TMR samples received at their laboratory between 2015 and 2020. These researchers detected ZEA (median = 23.2 μg/kg; maximum content = 452 μg/kg), DON (232 μg/kg; 3382 μg/kg), HT-2 toxin (18.2 μg/kg; 185 μg/kg), and nivalenol (40.8 μg/kg; 421 μg/kg) in all (100%) of them. T-2 toxin was found in 96.4% of the samples with a maximum content of 10.4 μg/kg. The consistent levels of these mycotoxins across the six studied years highlight the importance of routine on-farm control.

Some easy measures can be implemented in farms, such as storing feed for short periods and managing mycotoxins with feed additives. Classically, the mitigation of mycotoxin contamination in feed involves: (1) separate analysis of the raw materials present in the TMR [9]; (2) risk analysis of those raw materials [78]; (3) assessment of the use of mycotoxin adsorbents [34]; and (4) changes in the ingredients of the TMR. These measures allow us to obtain feasible control of mycotoxin contamination of raw feed but cannot assess the risk of a specific animal ingesting, storing and excreting [79] mycotoxins in milk.

Vaičiulienė et al. [2] observed that farms using imported feed (odds ratio = 3.40), feed storage >1 month (OR = 7.90), absence of use of antitoxins as a feed additive (OR = 2.30), and even intensive management systems (OR = 7.70) had a greater chance (p < 0.05) of consuming TMR contaminated with mycotoxins. These risk factors should be taken into consideration when designing and implementing a plan to prevent mycotoxin contamination in dairy farms.

The concept of estimated daily intake (EDI) was introduced in dairy production to evaluate the risk assessment of mycotoxins in farms according to animal daily ingestion and animal live weight [77]. The dietary exposure of cows to mycotoxins was previously measured as the average daily intake of mycotoxins per animal [54,56] but without considering the animal’s live weight, which can affect the threshold values of each mycotoxin acceptable for dairy cows. Biscoto et al. [77] observed the highest EDI value for FB (30.7 μg/kg bw/day) and for DON (30.7 μg/kg bw/day) but remained low for other analyzed mycotoxins, whereas AF presented the lowest value (0.3 μg/kg bw/day). These researchers considered that FB or DON alone did not represent a risk for the animals but were uncertain for the co-occurrence cases. The risk assessment using TMR samples can facilitate EDI determination. Furthermore, high EDIs can pose relevant concerns in public health other than animal health. An estimated scenario of significant risk (hazard quotient = 1.112) for children 4 years old consuming milk contaminated with ZEA (4.46 μg/L) was observed from a dairy farm presenting an EDI value of 260 μg/kg bw/day [78].

## 3 Conclusions

The concomitant number of raw materials present in diets is increasing, with different provenances worldwide, and bringing different risks to the farm that need to be accessed properly. The analysis of TMR can be a useful tool to monitor the risk of mycotoxin daily intake by the cow. On several occasions, it has been considered as the only practical way to screen the presence of mycotoxins coming from the different raw materials in dairy farms. Regular mycotoxin monitoring of TMR samples at critical periods, such as changes in raw feed, is advised to improve feed and food safety as well as animal health. Currently, we can observe the increasing effects of the presence of mycotoxins in animal feed due to the relevant double and multiple co-occurrences of the main mycotoxins AF, ZEA, DON, T2 toxin and FB, as well as by the underestimated presence and effect of emerging mycotoxins.

Further investigation should be considered to identify contaminated raw materials in the TMR and the implementation of a plan to minimize feed mycotoxin contamination on the dairy farm. Establishing EDIs values for a wide range of mycotoxins seems to be relevant to preventing health disorders in dairy farms.

## Funding statement

This work was partially supported by projects UIDP/CVT/00772/2020 (CECAV) and LA/P/0059/2020 (AL4AnimalS), funded by the 10.13039/501100001871Portuguese Foundation for Science and Technology (FCT).

## Data availability statement

No data was used for the research described in the article.

## Additional information

No additional information is available for this paper.

## CRediT authorship contribution statement

**Daniela Martins:** Writing – review & editing, Writing – original draft, Conceptualization. **Ana Lemos:** Writing – review & editing, Writing – original draft, Conceptualization. **João Silva:** Writing – review & editing, Writing – original draft, Conceptualization. **Marta Rodrigues:** Writing – review & editing, Writing – original draft, Conceptualization. **João Simões:** Writing – review & editing, Writing – original draft, Supervision, Conceptualization.

## Declaration of competing interest

The authors declare the following financial interests/personal relationships which may be considered as potential competing interests:Joao Simoes reports financial support, administrative support, and writing assistance were provided by 10.13039/501100001871Portuguese Foundation for Science and Technology. Ana Lemos reports a relationship with Animal Nutrition, DSM, The Netherlands that includes: employment. If there are other authors, they declare that they have no known competing financial interests or personal relationships that could have appeared to influence the work reported in this paper.

## Abbreviations

AF: Aflatoxins
BEA: Beauvericin
DON: Deoxynivalenol
EDI: Estimated daily intake
ENN: Enniatins
FB: Fumonisins
OTA: ochratoxin A
TMR: Total mixed ration
ZEA: Zearalenone
